# Imaging nodal volume and survival in oral tongue cancer with cervical lymph node metastasis

**DOI:** 10.1016/j.bjorl.2023.03.006

**Published:** 2023-03-21

**Authors:** Rattawut Wiengnon, Chakkapong Chakkabat, Napadon Tangjaturonrasme, Worawat Rawangban

**Affiliations:** a Chulalongkorn University, Faculty of Medicine, Department of Otolaryngology, Bangkok, Thailand; b Chulalongkorn University, Faculty of Medicine, Department of Radiology, Bangkok, Thailand

**Keywords:** Oral cancer, Lymphatic metastasis, Tumor volume, Imaging, Survival analysis

## Abstract

**Objectives:**

Oral tongue cancer is the most prevalent type of oral cavity cancer and presents the worst prognosis. With the use of TNM staging system, only the size of primary tumor and lymph node are considered. However, several studies have considered the primary tumor volume as a possible significant prognostic factor. Our study, therefore, aimed to explore the role of nodal volume from imaging as a prognostic implication.

**Methods:**

Medical records and imaging (either from Computed Tomography (CT) or Magnetic Resonance Imaging (MRI) scan) of 70 patients diagnosed with oral tongue cancer with cervical lymph node metastasis between January 2011 and December 2016 were retrospectively reviewed. The pathological lymph node was identified, and nodal volume was measured using the Eclipse radiotherapy planning system and was further analysed for its prognostic implications, particularly on overall survival, disease-free survival, and distant metastasis-free survival.

**Results:**

From A Receiver Operating Characteristic (ROC) curve analysis, the optimal cut-off value of the nodal volume was 3.95 cm^3^, to predict the disease prognosis, in terms of overall survival and metastatic-free survival (*p* ≤ 0.001 and *p* = 0.005, respectively), but not the disease-free survival (*p* = 0.241). For the multivariable analysis, the nodal volume, but not TNM staging, was a significant prognostic factor for distant metastasis.

**Conclusions:**

In patients with oral tongue cancer and cervical lymph node metastasis, the presence of an imaging nodal volume of ≥3.95 cm^3^ was a poor prognostic factor for distant metastasis. Therefore, the lymph node volume may have a potential role to adjunct with the current staging system to predict the disease prognosis.

**Level of Evidence:**

2b.

## Highlights

• In General, TNM staging which primary tumor and nodal volume is not included, been used to predict the prognosis of oral cavity cancer.
• Recent studies, primary tumor volume has increasing evidence for predict prognosis.
• We studied the role of nodal volume in oral tongue cancer for the survival outcomes.
• The result showed that nodal volume of ≥3.95 cm^3^ was a poor prognostic factor for distant metastasis.
• Nodal volume may use in adjunct with TNM staging to predict disease prognosis in oral tongue cancer.

## Introduction

Incidence of oral cavity cancer was increasing. In Thailand, the overall cancer mortality rate had been increasing in numbers over the years.^1^ Among them, oral cavity cancer is the seventh most common cancer in Thai male, and oral tongue is the most common site. The incidence of oral tongue cancer is 2.2 and 1.0 per 100,000 males and females, respectively. Overall 5-year survival rate of oral cavity cancer is 25.9%, while oral tongue cancer had the worst prognosis.^2^

In general, TNM staging system has been used to predict the disease prognosis, where size, number, and location of the primary Tumor (T), lymph Node (N) and the presence of distant Metastasis (M) are the primary considerations. However, in recent studies, the primary tumor volume has increasing evidence for its possible prognostic implication. Particularly in head and neck cancer, the primary tumor volume is a promising prognostic factor for predicting disease recurrence and distance metastasis.^3^, ^4^ Therefore, our study aims to explore the additional benefit of nodal volume from imaging on its prognostic implication together with current TNM staging system in oral tongue cancer.

## Methods

This retrospective cohort study was approved by the Institutional Review Board (IRB), Faculty of Medicine, Chulalongkorn University, IRB No.294/61. The protocol was performed in accordance with the declaration of Helsinki and its later amendments. Informed consent was waived due to an anonymous data extraction with no direct patient and public involvement in the study.

### Study participants

Medical records of patients diagnosed with oral tongue cancer and cervical lymph node metastasis at King Chulalongkorn Memorial Hospital between January 2011 and December 2016 were reviewed. The inclusion criteria were as follows: 1) Diagnosed with oral tongue cancer with cervical lymph node metastasis between January 2011 and December 2016; 2) Had the pre-operative Computed Tomography (CT) or Magnetic Resonance Imaging (MRI) scan compatible with the Eclipse application (Version 15.6.05, Varian company); and 3) Had the characteristics of lymph node metastasis including short-axis diameter of >1 cm, necrotic/cystic content, irregular border, and matted lymph node. The study excluded patients younger than 18 years old or non-Thais.

### Measures of lymph node volume

**Figure 1:**
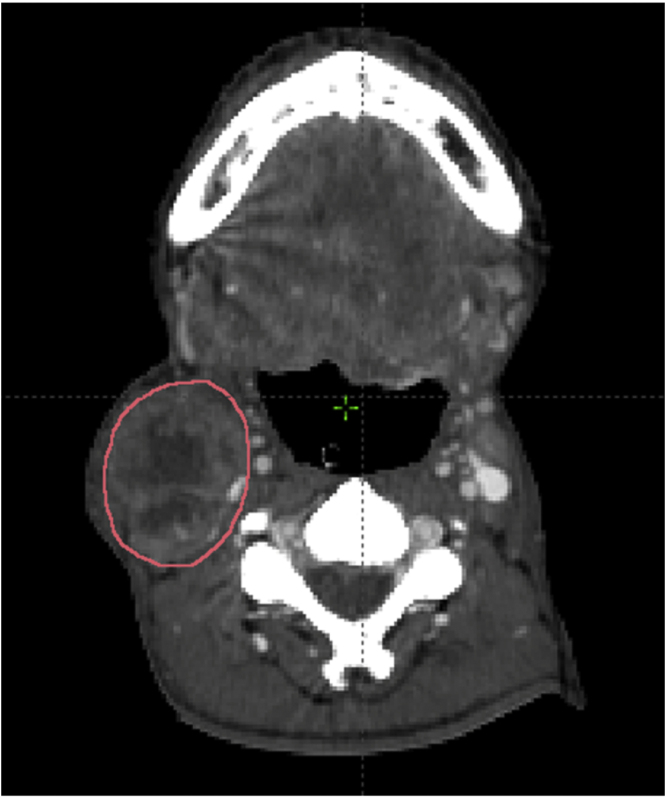
Contouring and volume measurement of the pathological lymph node in Eclipse radiotherapy planning system. External Beam Planning Volume = 75.99cm^3^ Equiv. Sphere Diam. = 5.3cm

The pathological lymph node was identified according to the inclusion criteria. Imaging data set was imported and reviewed in the Eclipse radiotherapy planning system. The lymph node was manually contouring each axial CT slice. The summation of the nodal volume was calculated using the volume computing function of the radiotherapy planning system (Fig. 1).

### Data collection

All demographics and clinical characteristic data of the patients, including sex, age, stage of the disease (The 7^th^ American Joint Committee on Cancer [AJCC] staging system),^5^ the nodal volume, the date of disease diagnosis, the date of metastasis diagnosis, the date of the death, aim of treatment and treatment modality were collected from King Chulalongkorn Memorial hospital’s electronic medical record. Survival data was retrieved online from national census database.

### Statistical analysis

**Figure 2:**
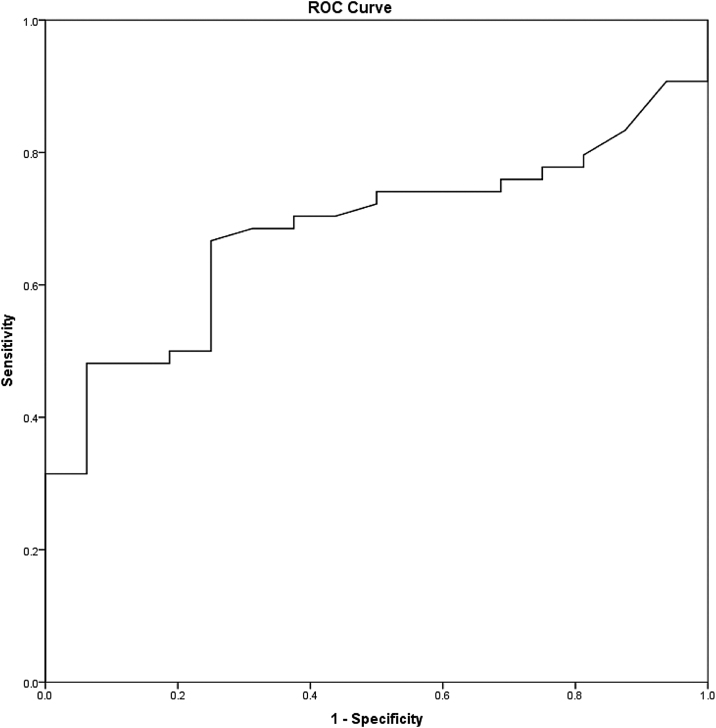
A Receiver Operating Characteristic (ROC) curve analysis of the optimal cut-off value to predict the mortality.

All statistical analysis was performed using IBM® SPSS® Statistic version 22. The Kolmogorov-Smirnov test was used to evaluate the probability distribution of the samples with the reference data. Categorical data were described as median and percentages. A Receiver Operating Characteristic (ROC) curve analysis was performed to identify the nodal volume's optimal cut-off value with the highest possible sensitivity and specificity (Fig. 2). For survival analysis, the Kaplan-Meier method and the log-rank test were used. The Cox proportional hazard model and the univariate and multivariate analysis were performed to evaluate the prognostic factors. Overall survival, disease-free survival, and distant metastasis-free survival were considered to determine the prognostic implications. The *p*-value of <0.05 was considered statistically significant.

## Results

Overall, 236 patients were diagnosed with oral tongue cancer between January 2011 and December 2016; however, 45 patients had no pre-operative CT or MRI scan imaging, 118 patients had no radiological pathologic lymph node, and three patients were non-Thais. Finally, 70 patients were included in the study.

Males were predominant (67.1%). The mean age was 58 years old, 62.8% of the patients were in stage IVA, and 70% of the histopathological results were well-differentiated Squamous Cell Carcinoma (SCCA). For overall survival analysis, the patients' median survival time was 13 months, the 1-year survival rate was 51%, and the 3-year survival rate was 24%.

Recurrence of the disease was found in 17 patients (24.2%), which the sites of recurrence were at oral tongue (14 patients) and cervical lymph node (3 patients). The distant metastasis was present in 19 patients, including lungs (12 patients), bones (4 patients), and other sites (3 patients).

The mean (SD) nodal volume was 5.31 (SD) cm^3^. In ROC curve analysis, the optimal cut-off value for the nodal volume was 3.95 cm^3^ (Area Under the Curve [AUC = 0.682], Sensitivity = 66.7%, and Specificity = 75%) (Fig. 2). Regarding the optimal nodal volume cut-off value, the patients were classified into 1) Small volume; the nodal volume of < 3.95 cm^3^ group (30/70 patients, 42.9%) and 2) Large volume; the nodal volume of ≥ 3.95 cm^3^ group (40/70 patients, 57.1%). The patients’ demographics and clinical characteristics are illustrated in Table 1.

**Table 1:** Demographic data and clinical characteristics of the patients.

Demographic data and clinical characteristics	LN volume < 3.95 cm^3^ (n = 30)	LN volume ≥ 3.95 cm^3^ (n = 40)
**Age** (years, min ‒ max)	56.5 (31‒87)	60 (23‒84)
**Gender**		
Female, n (%)	14 (46.67%)	8 (20%)
Male, n (%)	16 (53.33%)	32 (80%)
**T Stage**		
Early T, n (%)	17 (56.67%)	6 (15%)
Advance T, n (%)	13 (43.33%)	34 (85%)
**N Stage**		
N1, n (%)	18 (60%)	5 (12.5%)
N2, n (%)	11 (36.67%)	25 (62.5%)
N3, n (%)	1 (3.33%)	10 (25%)
**M stage**		
M0, n (%)	29 (96.67%)	36 (90%)
M1, n (%)	1 (3.33%)	4 (10%)
**Group stage**		
Stage III, n (%)	8 (26.67%)	3 (7.5%)
Stage IVA, n (%)	20 (66.67%)	24 (60%)
Stage IVB, n (%)	1 (3.33%)	9 (22.5%)
Stage IVC, n (%)	1 (3.33%)	4 (10%)
**Pathology**		
SCCA		
Well differentiated, n (%)	21 (70%)	21 (52.5%)
Moderately differentiated, n (%)	8 (26.67%)	17 (42.5%)
Poorly differentiated, n (%)	1 (3.33%)	2 (5%)
**Treatment**		
Surgical Upfront, n (%)	21 (70%)	19 (47.5%)
Non-surgical, n (%)	9 (30%)	21 (52.5%)

LN volume, Lymph Node volume; SCCA, Squamous Cell Carcinoma; T-stage, Tumor stage, N-stage, Lymph nNode stage, M-Stage, Distant Metastasis stage.

Additionally, the median (IQR) nodal volume of the patients in the dead group was significantly higher than the alive group (6.5 cm^3^ [2.49‒23.12] vs. 3.1 cm^3^ [1.9‒7.7], *p* = 0.043). The nodal volume of the patients with distant metastasis was likely to be larger than the patients with no metastasis (Table 2).

**Table 2:** The median lymph node volume of the patients.

Status (n)	LN Volume, median (IQR)	*p*-value
Dead (55)	6.5 (2.49–23.12)	0.043*
Alive (15)	3.1 (1.9–7.7)	
Recurrence (16)	4.45 (1.85–15.7)	0.690
No recurrence (54)	5.81 (2.49–18.9)	
Metastasis (14)	8.95 (4.6–20.1)	0.390
No metastasis (56)	4.15 (2.15–17.1)	

LN volume, Lymph Node volume.

* P value = < 0.05

### Survival outcome analysis

**Figure 3:**
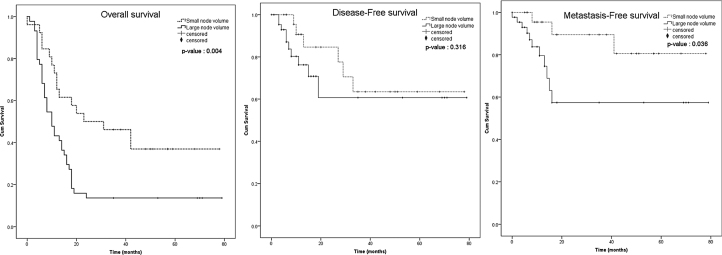
The Kaplan-Meier curves and the log-rank test of the overall survival, disease-free survival, and metastasis-free survival.

**Figure 4:**
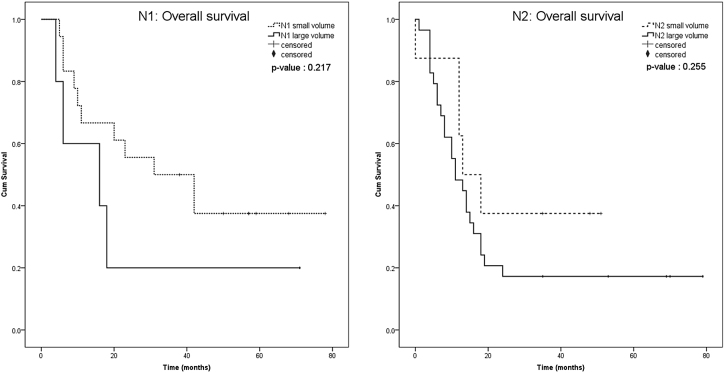
The Kaplan-Meier of the overall survival in each N-stage, stratum with nodal volume.

The Kaplan-Meier survival curve of both groups, including the patients with the nodal volume of <3.95 cm^3^ and ≥3.95 cm^3^, were identified and compared using the log-rank test. Overall survival and distant metastasis-free survival were significantly different between the nodal volume of <3.95 cm^3^ and ≥3.95 cm^3^ groups (*p* = 0.004 and *p* = 0.036, respectively), but not for the disease-free survival (*p* = 0.316) (Fig. 3). We performed subgroup analysis to compare the overall survival between smaller and larger nodal volume in the same N staging. The Kaplan-Meier survival curve obviously demonstrated trend of lower survival in larger nodal volume in N1 and N2 groups. Anyway, the different was not statistically significant (*p* = 0.217 in N1, *p* = 0.255 in N2) (Fig. 4). No analysis was done in N3 stratum due to most of them had large nodal volume.

From 2-year survival analysis, overall survival rate of patients with the nodal volume of <3.95 cm^3^ and ≥3.95 cm^3^ were 50% (95% CI 0.3130‒0.6612) and 12.5% (95% CI 0.0458‒0.2461), respectively. The metastasis-free survival rate of the nodal volume of <3.95 cm^3^ and ≥3.95 cm^3^ groups were 90% (95% CI 0.6707‒0.9763) and 48% (95% CI 0.2359‒0.6978), respectively, while for the disease-free survival rate were 82.5% (95% CI 0.5952‒0.9311) and 55.49% (95% CI 0.2426‒0.7827), respectively.

### Multivariate analysis

In univariate and multivariate analysis, the Cox proportional hazard model was applied to assess the association between the variables (e.g., age, sex, T-stage, N-stage, M-stage, TNM-stage, treatment options [surgery upfront or without surgery], and nodal volume [<3.95 cm^3^ and ≥3.95 cm^3^]), and the survival outcomes (e.g., overall survival, disease-free survival, and distant metastasis-free survival). Only the nodal volume was significantly associated with the distant metastasis-free survival (Adj HR = 4.45, *p* = 0.036, 95% CI 1.1‒17.94). The nodal volume was also likely to be associated with overall survival but not statistically significant (Table 3).

**Table 3:** Univariate and multivariate analysis of the factors associated with the survival outcomes.

Univariate analysis						
Variables	Overall survival		Disease-free survival		Metastasis-free survival	
	HR (95%CI)	*p*-value	HR (95%CI)	*p*-value	HR (95%CI)	*p*-value
**Age**	1.02 (1.01‒1.04)	0.046*	1.02 (0.99‒1.06)	0.183	1.03 (0.99‒1.07)	0.144
**Gender**	0.83 (0.47‒1.44)	0.503	1.06 (0.37‒3.06)	0.916	1.1 (0.34‒3.51)	0.873
**T Stage**						
1	Reference	1	Reference	1	Reference	1
2	1.75 (0.38‒7.99)	0.470	0.45 (0.07, 2.69)	0.382	11360.97 (0, 1)	0.942
3	7.11 (1.35‒37.4)	0.021*	1.71 (0.14, 20.3)	0.673	46721.01 (0, 1)	0.934
4	5.28 (1.26‒22.11)	0.023*	1.33 (0.28, 6.24)	0.720	52409.94 (0, 1)	0.933
**N Stage**						
1	Reference	1	Reference	1	Reference	1
2	1.74 (0.93‒3.25)	0.083	1.12 (0.39, 3.26)	0.829	1.82 (0.54, 6.1)	0.333
3	4.25 (1.84‒9.78)	0.001*	2.3 (0.44, 12.07)	0.325	3.43 (0.59, 19.89)	0.170
**M Stage**						
0	Reference	1	Reference	1	Reference	1
1	1.8 (0.71‒4.56)	0.215	0.04 (0, 775.69)	0.532	1.41 (0.18, 10.95)	0.740
**Staging**						
3	Reference	1	Reference	1	Reference	1
4	3.25 (1.28‒8.23)	0.013*	0.96 (0.3, 3.05)	0.950	4.61 (0.59, 35.81)	0.144
**Treatment surgery vs. no surgery**	0.49 (0.29‒0.84)	0.01*	1.5 (0.48‒4.68)	0.49	0.63 (0.22‒1.83)	0.394
**LN Volume ≥ 3.95 vs. <3.95 cm^3^**	2.53 (1.43‒4.48)	0.001*	1.82 (0.66‒5.05)	0.247	5.52 (1.49‒20.4)	0.01*

Multivariate analysis				
Variables	Overall survival		Metastasis-free survival	
	Adj HR (95% CI)	*p*-value	Adj HR (95% CI)	*p*-value
**Age**	1.02 (1‒1.04)	0.079	‒	
**Staging**				
3	Reference	1	Reference	1
4	2.34 (0.86‒6.34)	0.095	2.28 (0.26‒20.15)	0.459
**Treatment surgery vs. no surgery**	0.73 (0.41‒1.3)	0.291	1.05 (0.35‒3.15)	0.936
**LN Volume ≥ 3.95 vs. <3.95 cm^3^**	1.8 ( 0.97‒3.34)	**0.062**	4.45 ( 1.1‒17.94)	0.036*

CI, Confidence Interval; LN volume, Lymph Node volume; Adj HR, Adjusted Hazard Ratio; T-stage, Tumor stage; N-stage, Lymph Node stage, M-Stage, Distant Metastasis stage.

* P value = < 0.05

## Discussion

Oral tongue cancer is one of the worse prognoses in head neck cancer. We included only patients who had nodal metastasis, which make this cohort of purely advance stage cancer. Surgery upfront considers standard treatment follow by post-operative radiation or systemic therapy as indicated. However, with more extensive surgery especially in larger nodal volume group, half of patients refused the surgery and lead to somewhat lower survival compare with standard.

In this study, the prognostic implications of the volume of the lymph node, or the nodal volume, in oral tongue cancer were described for the first time. The 1-year survival rate in our study was 51%, which was slightly lower than the 1-year survival rate of oral cavity cancer reported by Muller P.^6^ The difference could be partially explained by the different population and ethnicity.

From A Receiver Operating Characteristic (ROC) curve analysis, the optimal cut-off level of the nodal volume of 3.95 cm^3^ illustrated the potential diagnostic capability of the nodal volume to determine the survival outcomes, including overall survival and distant metastasis-free survival (sensitivity = 65.5%, and specificity = 73.3%). In multivariate analysis, only the nodal volume, but not the TNM stage, was a prognostic factor for distant metastasis-free survival (Adj HR = 4.45, *p* = 0.036, 95% CI 1.1‒17.94).

In general, the TNM staging system is used to predict the disease prognosis. The staging system mainly focuses on the size of the primary tumor and its invasion to the adjacent organs. Apart from the primary tumor size, the role of the primary tumor volume, predicting the disease prognosis has been reported with increasing evidence. Strongin et al. indicated the primary tumor volume of >35 cm^3^, but not the TNM stage was associated with the poor prognosis for recurrence and distant metastasis.^5^ Lin CS et al. also revealed that the primary tumor volume was associated with the recurrent rate and the death rate.^7^ Anyway, due to retrospective study, this cohort used the 7^th^ American Joint Committee on Cancer (AJCC) staging system. Tumor thickness was not included.

In our study, the role of the nodal volume on its prognostic implications was explored and demonstrated the significant association between the nodal volume and the disease prognosis. The survival in the same Lymph Node (N) stage clearly different when categorized with nodal volume status. Even statistical non-significant, the trend to lower survival highlights the potential to consider the role of nodal volume in future study.

In multivariate analysis, the larger nodal volume (optimal cut-off volume = 3.95 cm^3^) was significantly associated with the likelihood of distant metastasis. The association between the nodal volume and the overall survival was also likely, but not statistically significant (Adj HR = 1.8, *p* = 0.062, 95% CI 0.97‒3.34). The non-significance may be partly due to the small number of patients. With the smaller nodal volume, the N-stage was likely to be lesser, and vice versa. Therefore, the patients in the groups classified by the nodal volume were not evenly distributed in terms of the N stage, thus affecting the statistical analysis. We cannot include others surgical risk factor parameter such as margin status, extranodal extension, perineural invasion, etc into multivariate analysis due to missing those data from non-surgical patients. This point needs future study to clarify all confounder that effect survival.

This study still has many limitations. Due to the retrospective design, some information was unavailable or missing, especially the pathologic findings such as depth of invasion, perivascular invasion, perineural invasion and marginal status so we did not use pathologic findings as the factors which may impact the outcomes. The measurement technique of the nodal volume, in which only the Computed Tomography (CT) or Magnetic Resonance Imaging (MRI) scan imaging of the lymph node that contained the characteristics of possible metastasis were included. Therefore, the lymph node of size <1 cm, which may be metastatic, was excluded from the measure, which affected the total nodal volume. Due to the single-center study with limited number and distributions of the patients, further multi-center study with more number and distributions of the patients may provide more precise data and warrant the subgroup analysis, including comparing the nodal volume in the patients with the same N-stage, to define the prognostic implications of the nodal volume and the N-stage.

## Conclusions

In patients with oral tongue cancer and cervical lymph node metastasis, the presence of a nodal volume of ≥3.95 cm^3^ was a poor prognostic factor for distant metastasis. Therefore, the lymph node volume from the current imaging protocol may be useful in adjunct with the current staging system to predict the disease prognosis.

## Funding

No funding.

## Conflicts of interest

The authors declare no conflicts of interest.
